# Perfluorooctanoic acid (PFOA) exposure in relation to the kidneys: A review of current available literature

**DOI:** 10.3389/fphys.2023.1103141

**Published:** 2023-01-26

**Authors:** Dongge Liu, Shuqi Yan, Pingwei Wang, Qianqian Chen, Yanping Liu, Jiajing Cui, Yujun Liang, Shuping Ren, Ying Gao

**Affiliations:** ^1^ Department of Occupational and Environmental Health, School of Public Health, Jilin University, Changchun, China; ^2^ Department of Endocrinology, The First Hospital of Jilin University, Changchun, China

**Keywords:** perfluorooctanoic acid, kidney, pfoa, PFAS, CKD, EGFR, renal tumors

## Abstract

Perfluorooctanoic acid is an artificial and non-degradable chemical. It is widely used due to its stable nature. It can enter the human body through food, drinking water, inhalation of household dust and contact with products containing perfluorooctanoic acid. It accumulates in the human body, causing potential harmful effects on human health. Based on the biodegradability and bioaccumulation of perfluorooctanoic acid in the human body, there are increasing concerns about the adverse effects of perfluorooctanoic acid exposure on kidneys. Research shows that kidney is the main accumulation organ of Perfluorooctanoic acid, and Perfluorooctanoic acid can cause nephrotoxicity and produce adverse effects on kidney function, but the exact mechanism is still unknown. In this review, we summarize the relationship between Perfluorooctanoic acid exposure and kidney health, evaluate risks more clearly, and provide a theoretical basis for subsequent research.

## 1 Introduction

Perfluorinated and polyfluoroalkyl substances (PFAS), also called perfluorinated compounds (PFCs), are perfluorinated alkyl and polyfluoroalkyl substances containing one or more C atoms, mainly include perfluorooctanoic acid (PFOA), perfluorooctane sulfonate (PFOS) and many other compounds (Kato et al., 2011). Since the 1960s, PFAS has been widely used in production. The widespread use of PFAS makes it present in soil, air and water in all regions of the world, even in remote polar regions (Giesy and Kannan, 2002). Although there may be demographic, geographic, and temporal differences in exposure PFAS is prevalent in the general population (Yeung et al., 2006; Fromme et al., 2007; Lau et al., 2007).

Perfluorooctanoic acid (PFOA), as one of the prominent environmental pollutants in the perfluoroalkyl group, can be found in almost all populations. PFOA levels in plasma from male and female American Red Cross blood donors ranged from 1.1 to 3.4 ng/mL, higher than other measured PFCs (Olsen et al., 2017). Another study conducted in Poland showed a PFOA concentration of 0.67–12.56 ng/mL in the plasma of 429 Polish citizens (Goralczyk et al., 2015). Serotesting of healthy women born between 1934 and 1967 in Sweden found PFOA concentrations between 1.78 and 5.51 ng/mL (Axmon et al., 2014). In a Greek study, serum PFOA levels were 1.70 ng/mL in women and 3.14 ng/mL in men (Vassiliadou et al., 2010). Germany reported serum PFOA levels in the general population ranging from 1.7 ug/L to 39.3 ug/L (Midasch et al., 2006). Similarly, 10 types of PFAS were found in the plasma of women of childbearing age in Shanghai, China, which were detectable in 98% of samples, with the highest concentration of PFOA at a median of 13.83 ng/mL (Zhou et al., 2019). In a study comparing PFO levels in different countries, PFOA was found to be the second most abundant perfluorinated chemical in serum samples from the United States, Colombia, Brazil, Belgium, Italy, Poland, India, Malaysia and South Korea (Kannan et al., 2004).

Given that human exposure to PFAS levels is related to drinking water concentrations (Jin et al., 2009; Garnick et al., 2021), EU members, such as Sweden, set the recommended maximum exposure level at 90 ng/L for the sum of the seven PFAS in drinking water, including PFOA, according to the National Food Agency (Banzhaf et al., 2017). The EPA recently issued a lifetime drinking water health recommendation of 0.07 ug/L for PFOA, PFOS, or both (EPA and US, 2016a; EPA and US, 2016b). Correspondingly, seven United States. states have also developed their own PFOA water guideline levels, ranging from 13 to 1000 ng/L (Cordner et al., 2019).

The half-life of PFOA in humans can be up to several years (Li et al., 2018). Once absorbed by the human body, it can enter human serum, breast milk, liver and kidney and accumulate (Awad et al., 2020; Gustafsson et al., 2022). Animal studies show that after PFOA exposure, the highest concentration is found in the main target organ, the kidney, followed by the liver and lungs (Cui et al., 2009). Contrary to the fact that experimental animals can excrete a large amount of urine (Vanden Heuvel et al., 1991; Vanden Heuvel et al., 1992a), PFOA is not metabolized in the human body and is discharged from the kidney without biotransformation. Due to reabsorption by the kidneys, PFOA is reabsorbed into the kidneys, causing ongoing effects on the kidneys (Harada et al., 2005; Lau et al., 2007; Loccisano et al., 2011).

**FIGURE 1:**
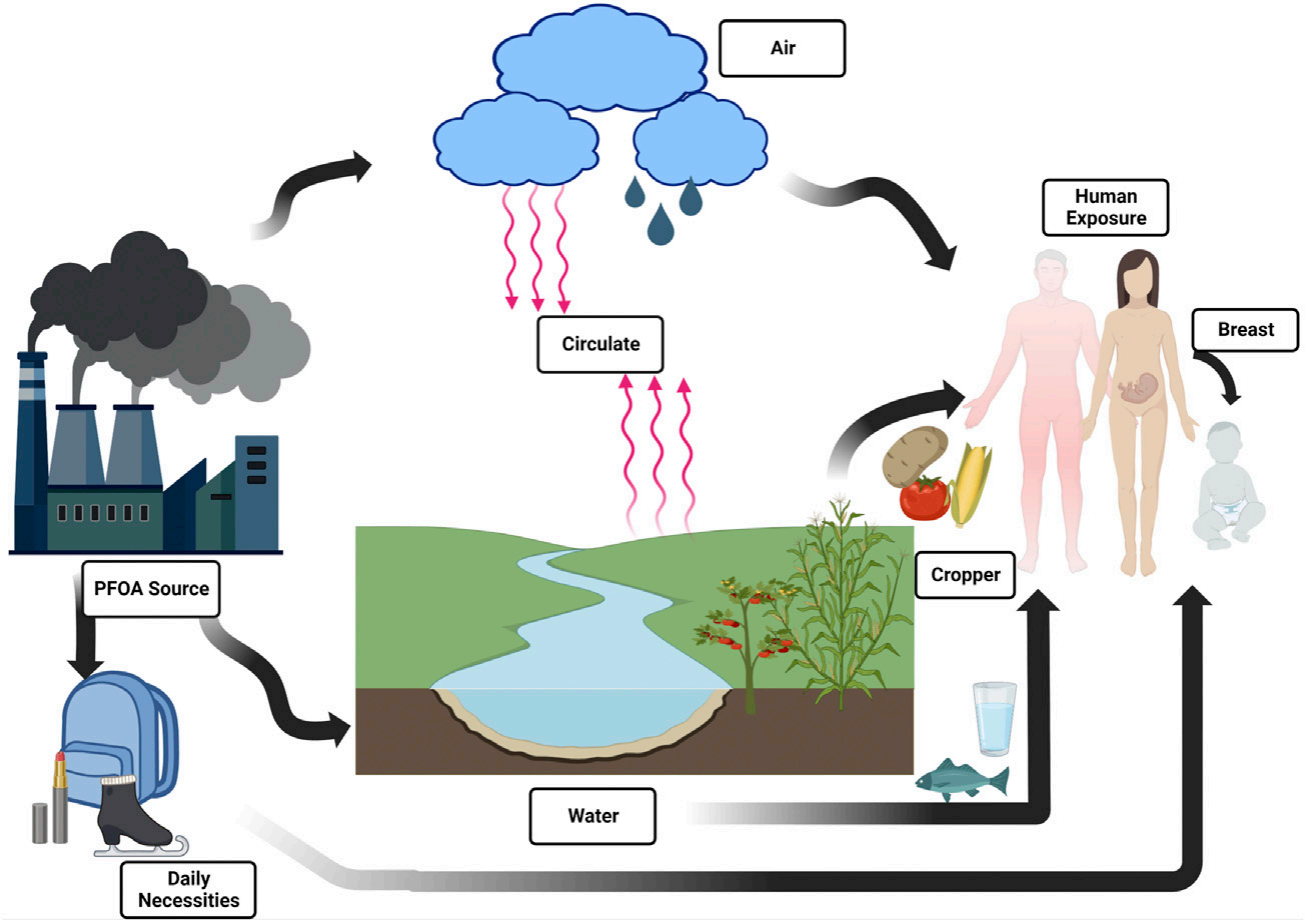
Overview of PFOA exposure pathways for different human populations outside of occupational settings.

Therefore, it is of great significance to summarize the adverse effects of PFOA on kidney and its related mechanisms. We searched the studies on the relationship between PFOA and kidney, including cell, animal experiments and epidemiological surveys to summarize the effects of PFOA on kidney (Figure 1).

## 2 Overview of perfluorooctanoic acid (PFOA)

Perfluorooctanoic acid (PFOA) is a synthetic chemical consisting of perfluorooctanoic eight carbon chain with carboxylic acid functional group, which is an industrial by-product of manufacturing commercial polymers, present in the serum of residents in most industrialized countries (Buck et al., 2011). In the United States, a small amount of measurable PFOA has been detected in the serum of more than 95% of the population (Calafat et al., 2007; Kato et al., 2011). However, the mobility, persistence and widespread use of PFOA have led to the pollution of global drinking water, and PFOA levels in the drinking water of more than 6 million American residents exceeds the lifelong health recommendations (Hu et al., 2016). Similarly, because of the increase of large-scale production in developing countries such as China, the global exposure to PFOA is still high (Li et al., 2021). From 2004 to 2012, China produced 480 tons of PFOA, and the cumulative environmental emissions reached 250 tons. The annual output of PFOA in China increased to 150 tons in 2013, making China the largest PFOA emitter in the world (Li et al., 2015). Perfluorooctanoic acid and its related compounds are proposed to be included in the Convention in 2019, and manufacturers in many countries have completed extensive phase out (UNEP, 2019). Due to the large-scale production and extensive use of PFOA, PFOA can be detected in almost all environmental media, including surface water, groundwater, drinking water, coastal seawater, sludge, sediment and soil. PFOA can be found in surface water samples in China (Chen et al., 2016; Huang et al., 2021; Podder et al., 2021).

Similarly, various types of PFAS have been found in drinking water samples collected at several European locations. For example, PFOA was found in 33% of samples in Brazil (Schwanz et al., 2016). Among the drinking water samples collected in 79 cities in China in 2017, at least one PFAS was detected in all samples, mainly PFOA and PFBA (perfluorobutyric acid) (Li et al., 2019). Epidemiological studies show that compared with adults, drinking water exposure to PFOA results in higher serum levels in infants (Post et al., 2012).

PFOA is highly stable, hydrophobic and oil repellent, and will not undergo photolysis and biodegradation (Kato et al., 2011). From protective coatings for textiles, leather, carpets and paper to pesticides, paints and cosmetics, as well as fire foam, hydraulic oil, wax and polishing agents (Lau et al., 2007; Ericson et al., 2008), PFOA is almost everywhere (Wang et al., 2017). Human PFOA exposure occurs in different ways. Most adults are exposed to PFOA mainly through drinking water and food (Haug et al., 2011; Shin et al., 2011; Bartell, 2017). Breast milk is considered to be the main contact route for infants, and PFOA can easily cross the placental blood barrier to reach the developing fetus (Haug et al., 2011; Rovira et al., 2019). Inhalation is the main exposure route for production workers. Some interior decoration workers can be exposed by ingesting household dust or inhaling PFOA precursors in indoor air (Sunderland et al., 2019). In some cases, dermal exposure may be an important route of contact, and perfluorooctanoic acid may be absorbed by the skin (Franko et al., 2012).

Currently, PFOA exists in almost everyone’s blood, liver and kidney (Kannan et al., 2004). The accumulation of PFOA in animals leads to a variety of toxicity, such as nephrotoxicity, immunotoxicity, neurotoxicity, developmental toxicity, hepatotoxicity, genotoxicity, pancreatic toxicity, cardiotoxicity, and thyroid toxicity (Yao and Zhong, 2005; Dewitt et al., 2008; Johansson et al., 2008; Qazi et al., 2010; Jiang et al., 2012; Coperchini et al., 2017; Song et al., 2018; Stanifer et al., 2018; Abudayyak et al., 2021). PFOA can also cause pregnancy diabetes and a variety of cancers, such as kidney cancer, testicular cancer, liver cancer, breast cancer, etc (Wang et al., 2018; Steenland and Winquist, 2021). PFOA exposure is reported to be positively related to asthma and asthma related biomarkers in Taiwan children (Fairley et al., 2007; Dong et al., 2013). PFOA will increase the levels of serum immunoglobulin E (IgE) and enhance the response of IgE to environmental allergens (Fairley et al., 2007; Dong et al., 2013). American children with high serum PFOA levels were found to be more likely to suffer from attention deficit hyperactivity disorder (ADHD) (Hoffman et al., 2010). In addition, PFOA exposure also has a significant impact on adult immunity (Pilkerton et al., 2018).

It is reported that PFOA can reach drinking water wells through polluted groundwater migration, and can also enter groundwater from the air discharge of nearby industrial facilities, or even deposit into soil from the air and migrate to groundwater through the soil (Davis et al., 2007). The use of granular activated carbon in water filtration systems has been proven to be effective in reducing serum PFOA concentrations in exposed populations (Bartell et al., 2010). However, even continuous exposure to relatively small amounts of PFOA will still lead to physical burden, which may increase the risk of adverse health effects (Post et al., 2012).

## 3 The relationship between perfluorooctanoic acid (PFOA) and kidney

PFOA has been used in many industrial and commercial applications for more than 50 years since the 1950s, and is one of the most popular perfluoroalkyl compounds (PFC) (Prevedouros et al., 2006). Different from the lipophilicity of other persistent organic pollutants, the polar hydrophobicity of PFOA results in an increase in the affinity of binding with proteins rather than lipids. Once PFOA enters the human body, it will preferentially adhere to proteins, readily accumulating in biological tissues and organs with high protein content, such as kidney, liver and serum (Vanden Heuvel et al., 1992b; Jones et al., 2003; Weiss et al., 2009). Kidney is considered a target of PFOA, and the biological accumulation of PFOA in the human body is partly due to its slow renal elimination. Kidney is the main organ for excreting metabolic wastes of the body, with reabsorption effects (Han et al., 2012). Perfluorooctanoic acid from other organs and blood will be transported to the kidney through circulation due to its metabolic characteristics (Loccisano et al., 2011).

PFOA exposure is also associated with elevated levels of homocysteine (HCY) and uric acid (UA) (Shankar et al., 2011a; Min et al., 2012). Healthy kidneys play an important role in the clearance and metabolism of homocysteine, and the decrease of renal function will be accompanied by the increase in homocysteine level (Friedman et al., 2001). Similarly, the kidney also plays a leading role in the excretion of uric acid. The kidney excretes about 70% of uric acid. The decline of renal function will cause the decrease of uric acid excretion and then lead to the increase of uric acid (Yanai et al., 2021). It is found that histological and cellular changes in renal tubules have been observed in several animal models due to PFOA exposure (Stanifer et al., 2018). Renal hypertrophy, tissue proliferation and microvascular disease developed in rats exposed to high levels of PFOA (Cui et al., 2009). Epidemiological studies have shown that the development of various kidney diseases is associated with PFOA exposure (Shankar et al., 2011a; Gong et al., 2019). Rashid et al. revealed that epigenetic changes caused by acute PFOA exposure may affect fibroblast activating genes, leading to renal fibrosis (Rashid et al., 2020).

The analysis of six wild fishes collected from edible fishes in Taihu Lake shows that the highest content of perfluoroalkyl acids (including PFOA) is in the kidneys and livers of fishes. The filtration rate of long-chain perfluoroalkyl substances in the kidney may be different from that of short chain perfluoroalkyl substances, which leads to their preferential accumulation in the kidney (Chen et al., 2021). Consumption of food, especially animal food such as aquatic products, is considered as the main way for humans to contact PFOA (Haug et al., 2010). Because of the physical and chemical properties and toxicity of perfluorooctanoic acid, it is particularly important to summarize the adverse effects of perfluorooctanoic acid on the kidney.

### 3.1 Difference of renal elimination in half life of perfluorooctanoic acid

The half-life of PFOA varies greatly among different species. The biological half-life (t 1/2) in male rats is 6 days, and that in female rats is about 1 h (D'Eon J and Mabury, 2010), and the excretion rate of PFOA in female rats is higher than that in male rats (Pizzurro et al., 2019). More than 70% elimination occurs in urine of female rats within 24 h, and less than 10% in male rats (Hanhijarvi et al., 1982; Kudo et al., 2001; Ohmori et al., 2003; Lau et al., 2006). It was observed that at the end of a 6-h exposure to PFOA, the plasma level of PFOA in male rats was almost twice that of female rats (Hinderliter et al., 2006). The estimated half-life of PFOA in humans ranges from 1.2 to 14.9 years (Olsen et al., 2007; Bartell et al., 2010; Brede et al., 2010; Loccisano et al., 2011; Yeung et al., 2013; Zhang et al., 2013; Worley et al., 2017; Xu et al., 2020; Xu et al., 2021; Li et al., 2022a). Many of these estimates do not take into account background or persistent PFOA exposure, which may result in a greater PFOA half-life than the actual half-life (Russell et al., 2015; Bartell, 2018). The PFOA half-life of about 1.5 years seems to be the most reliable estimate after deducting background exposure (Xu et al., 2020). The half-life discrepancy is mainly due to differences in renal clearance rates (CLR) (Kudo et al., 2002; Butenhoff et al., 2004; Andersen et al., 2006). It is reported that renal clearance is species- and gender-dependent (Han et al., 2012). In humans, there is no significant gender difference in the half-life of PFOA. However, the clearance rate of rat PFOA is largely gender-dependent (Kudo and Kawashima, 2003; Kennedy et al., 2004).

Renal clearance, including glomerular filtration, tubular secretion and reabsorption, is the most critical process to determine the systemic clearance rate of PFOA (Han et al., 2012). The diversity of proximal tubular organic anion transporters and the possibility of genetic variation indicate that the longer elimination half-life of perfluorooctanoic acid in humans compared with other species may be due to the differential expression of organic anion transporter genes, which may be related to the low level of PFOA transported to urine or increased renal tubular reabsorption (Kudo et al., 2002; Eraly et al., 2004; Ljubojevic et al., 2004).

In humans, PFOA is filtered freely in the glomerulus, secreted actively from blood to urine through proximal tubular cells, and then almost completely absorbed back into the blood (Loccisano et al., 2011). Since renal excretion depends partly on glomerular filtration, a higher glomerular filtration rate (eGFR) contributes to an increase in urinary excretion and a decrease in half-life. It can be understood that the half-life of PFOA of prepubertal children is 45%–60% shorter than that of people over 50 years old. Higher urinary excretion and serum ratio are related to higher elimination rate, which means that a higher proportion of PFOA is excreted through urine, thus reducing the half-life of PFOA in the body (Li et al., 2022a).

In addition, the affinity of PFOA to organic anion transporters also affects their excretion. For example, organic anion transport proteins (Oats/OATs) and organic anion transport peptides (Oatps/OATPs) have been identified as responsible for PFOA excretion and reabsorption in human and rat renal tubules (Yang et al., 2009; Weaver et al., 2010; Yang et al., 2010). The use all capital letters indicates human transporters; use of a capital letter followed by a small letter indicates animal transporters (Armitage et al., 2017).

Specific transporters (including organic anion transporters Oat1 and Oat3, Oat4 and urate transporter1 (Urat1) and organic anion transporter polypeptide (Oatp1a1), all contribute to the reabsorption of PFOA from renal filtrate in proximal tubules (Hinderliter et al., 2006; Nakagawa et al., 2008; Weaver et al., 2010). The existence and activity of these transporters are also considered as explanations of the huge difference in the elimination of PFOA between the sexes of mice, because it is known that the expression of many renal transporters is controlled by sex hormones, especially testosterone (Morris et al., 2003; Ljubojevic et al., 2007; Cheng and Klaassen, 2009). Inhibition of renal organic anion transport by probenecid can effectively reduce the renal clearance of PFOA in female rats to the same level as that in male rats (Kudo et al., 2002).

**FIGURE 2:**
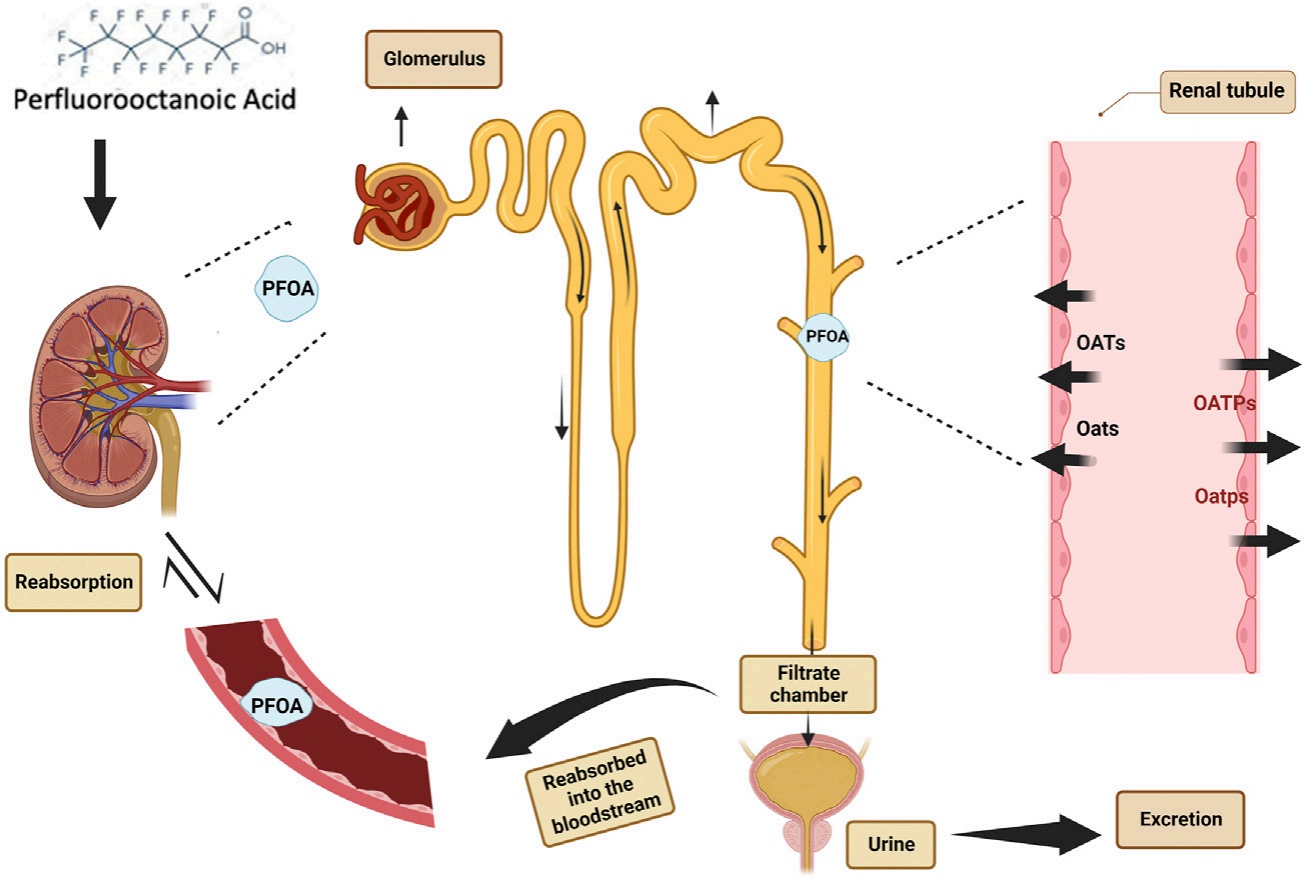
Difference of renal elimination in half life of perfluorooctanoic acid.

The renal organic anion transport system is an important system to promote the daily elimination of toxic compounds and the reabsorption of useful anion metabolites. Both human and rat OAT1 (Oat1) and OAT3 (Oat3) can transport perfluorooctanoic acid, but neither of the two species utilize OAT2 (Oat2) as transporters. As far as perfluorooctanoic acid transport is concerned, OAT1 (Oat1) and OAT3 (Oat3) transporters have similar transport activities in human and rat (Nakagawa et al., 2008). In conclusion, the study shows that PFCA (perfluoroalkyl carboxylic acid) with different chain lengths (including PFOA) is the substrate of two kinds of basolateral transporters, Oat1, Oat3 and top transporter Oatp1a1 in rat proximal tubular cells. Together, these basolateral membrane transporters transport PFOA from blood to proximal tubular cells and promote renal secretion (Buist et al., 2002; Weaver et al., 2010) (Figure 2).

#### 3.1.1 Perfluorooctanoic acid (PFOA) and glomerular filtration rate

The main function of the kidney is to filter blood with the glomerulus. Therefore, glomerular filtration rate (eGFR) is the most important marker of renal function for evaluating renal diseases. The glomerular filtration rate (eGFR) can be divided into the following stages: GF- 1, (eGFR>90 mL/min/1.73 m^2^); GF-2 (eGFR 60–89 mL/min/1.73 m^2^), GF-3A (eGFR 45–59 mL/min/1.73 m^2^), and GF-3B/4 (eGFR 15–44 mL/min/1.73 m^2^) (Inker et al., 2014).

Three cross-sectional studies reported an inverse correlation between PFOA and eGFR. Watkins et al. examined the correlation between eGFR and PFOA concentrations in children and adolescents in the C8 health program (n = 9660) and showed that a decrease in eGFR was associated with an increase in PFOA serum concentration (Watkins et al., 2013). (Kataria et al., 2015) The results of another cross-sectional study of NHANES adolescents (n = 1960) from 2003 to 2010 confirmed and amplified previous findings (Watkins et al., 2013). Adolescents with the highest PFOA concentrations had a reduction in eGFR of 4.37 mL/min/1.73 m^2^ (95% CI: −9.15 to 0.40) (Kataria et al., 2015). Community studies also found (*n* = 69,030) that both PFOA and PFOS were negatively correlated with eGFR (Conway et al., 2018). However, another cross-sectional study showed the opposite result (*n* = 1612), with PFOA positively correlated with higher eGFR (Wang et al., 2019).

These conflicting observations may be due to differences in study design, population, and PFAS exposure levels, cross-sectional studies cannot judge true causation, and findings may be attributed to reverse causation (Dhingra et al., 2017). It is recommended to use measured and simulated serum PFOA separately compared with eGFR, which can aid in interpreting the results more scientifically (Dhingra et al., 2017), or prospective studies to confirm this association. In a prospertive study (*n* = 875), higher baseline PFOA plasma concentrations were associated with lower eGFR, consistent with the conclusions of previous studies (Watkins et al., 2013; Kataria et al., 2015; Conway et al., 2018). Importantly, it was followed for about 14 years, and the results were robust, with prospective assessment and reverse causality testing of exposure and eGFR (Lin et al., 2021).

Other studies have provided us with new perspectives. An analysis of data from the National Health and Nutrition Examination Survey (*n* = 6,844) of adults over 20 years of age in the United States challenged the hypothesis that PFAS followed a one-way association throughout the renal function phase, concluding that there was a general inverted U-shaped association between PFOA and decreased eGFR in advanced renal disease, with the inflection point at GF-3A (Jain and Ducatman, 2019a), likewise, this result was verified in another study (Jain, 2021). Possible physiological explanations are related to the complex and slow excretion of PFAS in humans, where the balance between glomerular secretion and reabsorption can significantly affect serum concentrations of poisons (Loccisano et al., 2011). Another reason may be due to the interaction between age and kidney function (Weinstein and Anderson, 2010).

#### 3.1.2 Perfluorooctanoic acid (PFOA) and uric acid

Uric acid is a major biomarker of increased risk of kidney disease (Obermayr et al., 2008). Occupational workers are usually exposed to high levels of PFOA. In two cross-sectional studies of chemical workers, PFOA was found to be significantly associated with elevated uric acid (Sakr et al., 2007; Costa et al., 2009), in time, the two change together. Another case-control study of 40 occupational workers and 52 ordinary people showed that exposure to PFAS (including PFOA) caused the upregulation of plasma hypoxanthine, which could be oxidized into uric acid in the human body (Lu et al., 2019). Six cross-sectional studies on adults report that PFOA exposure is positively correlated with uric acid (Steenland et al., 2010; Shankar et al., 2011a; Gleason et al., 2015; Jain and Ducatman, 2019b; Zeng et al., 2019; Scinicariello et al., 2020). Scinicariello et al. first reported the association between serum PFAAs (perfluoroalkyl acids) and self-reported gout diagnosis in adults (*n* = 4,917). They found that the higher the quartile values of PFOA, PFNA (perfluoronanoic acid) and PFHxS (Perfluorohexane sulfonic acid), the higher the probability of hyperuricemia. The results showed that exposure to PFAA (including PFOA) may be a risk factor for hyperuricemia and gout (Scinicariello et al., 2020). The level of perfluoroalkyl chemicals is related to uric acid in both the general population and the highly exposed population. In the PFOA and uric acid study conducted among 54,951 adult community residents in Ohio and West Virginia, the residents affected by high exposure to PFOA (median 27.9 ng/mL) had higher serum PFOA levels and higher prevalence of hyperuricemia (Steenland et al., 2010). In the (Shankar et al., 2011a) (*n* = 3,883) and (Gleason et al., 2015) (*n* = 4,333, median of PFOA is 3.7 ug/L) studies, the low exposure population to PFOA and uric acid also showed a strong correlation.

Two cross-sectional studies stratified renal function (Zeng et al., 2019). In China, the association between serum PFAS isomers and serum uric acid was studied in 1612 participants. There was a positive correlation between serum PFAS isomers and uric acid, especially PFOA. The author explained that the correlation between total PFOA and hyperuricemia seemed to be driven by the isomer Sb PFOA (Zeng et al., 2019). Another study (Jain and Ducatman, 2019b) (*n* = 6,844) showed that, for GF-2 in men, PFOA was positively correlated with uric acid after stratification by renal function, and it was found that all five PFAS were positively correlated with uric acid in women.

A cohort study conducted a 10-year trend analysis of 13 major PFAS in 786 adults in South Korea. It was found that uric acid was positively correlated with PFAS (including PFOA) levels (Seo et al., 2018). PFOA level has been proved to be positively correlated with hyperuricemia in adults, but the evidence of correlation in children is still limited. The relationship between serum PFOA and PFOS levels and hyperuricemia was examined in a representative sample of American children (*n* = 1,772). The cross-sectional study results showed that serum perfluoroalkyl chemistry levels were significantly related to hyperuricemia in children, even under the low background exposure level of the general population in the United States (Geiger et al., 2013). In investigating the risk of hyperuricemia associated with perfluoroalkyl substances (PFASs) in children in Taiwan, China, we found that perfluorooctanoic acid was positively correlated with serum uric acid level, and in all PFASs analysis, only PFOA showed a significant impact on the increase of hyperuricemia level, but when stratified by sex, the association between serum PFOA and uric acid level was only obvious in boys, and further research is needed to clarify these links (Qin et al., 2016). In a cross-sectional study of an adolescent population, the correlation between PFOA and uric acid has diverged. A study found that PFOA was related to an increase of uric acid level in healthy adolescents (*n* = 1690) (Kataria et al., 2015). However, the study did not find a correlation between PFOA and serum uric acid, which may be due to the fact that the study population (*n* = 644) is composed of teenagers and young people with abnormal urine analysis (Lin et al., 2013).

The correlation strength between PFOA and uric acid is generally moderate. Since most studies are cross-sectional studies, the results may be limited by reverse causality. Some mechanisms can be applied to reverse causality, for example, PFOA and uric acid can share the renal transport system, and the renal transport system can control the excretion of each substance (Eraly et al., 2008; Nakagawa et al., 2008). Therefore, if the level of PFOA increases, the excretion of urate may decrease, so the level of blood urate may increase secondarily. However, whether this shared transporter hypothesis is related to humans remains a matter of speculation, so prospective research and human research are needed to comprehensively judge the results (Eraly et al., 2008).

#### 3.1.3 Perfluorooctanoic acid (PFOA) and chronic kidney disease

Chronic kidney disease (CKD) is defined as glomerular filtration rate below 60 mL/min/1.73 m^2^ for 3 months or more (Levey et al., 2005). CKD has become a major public health problem in developing and developed countries. In China, CKD affects about 119.5 million people, with a total prevalence rate of 10.8% (Zhang et al., 2012). The most commonly used markers for evaluating CKD are estimated glomerular filtration rate (eGFR), urinary albumin/creatinine ratio (ACR) and urinary albumin excretion rate (AER) (Jha et al., 2013; Das et al., 2016).

Epidemiological studies have shown that at different levels of human chronic exposure, PFOA is also associated with several risk factors or potential biomarkers of chronic kidney disease, including elevated total cholesterol and low-density lipoprotein (LDL) cholesterol (Steenland et al., 2009; Nelson et al., 2010), elevated uric acid level (Steenland et al., 2010; Shankar et al., 2011a), increased prevalence of insulin resistance and metabolic syndrome (Lin et al., 2009), elevated homocysteine level and elevated blood pressure (Friedman et al., 2001). Increased risk of hypertension is a strong predictor of CKD, however, this finding has not been confirmed in highly exposed populations (Winquist et al., 2013).

Although there is a large amount of evidence that PFOA poses a new health threat to kidneys, epidemiological studies have not yet confirmed the relationship between PFOA and CKD (Stanifer et al., 2018). Three cross-sectional studies found a positive correlation between CKD and measured PFOA (Watkins et al., 2013). Whether children younger than 18 years old under the high exposure background in the United States (*n* = 9,660) (Xie et al., 2022), or adolescents under the high exposure background in China (*n* = 1,612) (Shankar et al., 2011b), or the general population under the relatively low exposure background level in the United States (*n* = 5,717), the serum PFOA level is positively related to chronic kidney disease. However, another cross-sectional study of adults in Shenyang, China found that most PFAS was positively correlated with CKD, except for PFOA and PFDA (perfluorodecanoic acid) (Wang et al., 2019).

A mortality study showed an association between PFOA exposure estimated from a work exposure matrix and mortality from chronic kidney disease (Steenland and Woskie, 2012). However, another study on the incidence rate of diseases in a DuPont occupational cohort (*n* = 3,713) found no correlation between the estimated cumulative PFOA serum level and the incidence rate of chronic kidney disease (Steenland et al., 2015). Cross-sectional studies may be affected by reverse causality, while mortality studies are subject to greater disease misclassification than incidence rate studies of non-fatal diseases. Therefore, two prospective studies can provide us with new strong evidence. Radhika Dhingra et al. conducted a longitudinal analysis of chronic kidney disease among adults exposed to PFOA (*n* = 32,254) in the Central Valley Community cohort in Ohio and found that there was no relationship between PFOA exposure and CKD. No significant difference was found between the two in either retrospective or prospective analyses (Dhingra et al., 2016). It is worth noting that the participants were at a high level of PFOA exposure (median 28.2 ug/L), and the outcome considered the incidence rate of CKD rather than the mortality of CKD, which avoided the non-standard recording of CKD causes of death. In another prospective cohort analysis (*n* = 967) on the association between serum PFOA and PFOS levels in patients with type 2 diabetes and the risk of chronic kidney disease, CKD events were not related to PFOA (Li et al., 2022b), which was consistent with the longitudinal analysis of non-diabetes subjects in the central Ohio Valley community (Dhingra et al., 2016).

## 4 Perfluorooctanoic acid (PFOA) and renal tumors

Renal cancer is worrisome, and the relationship between PFOA exposure and renal cancer has drawn different conclusions in four worker cohort mortality studies. There was no association between PFOA and renal cell carcinoma in studies (Leonard et al., 2008; Raleigh et al., 2014). In several studies, PFOA exposure was found to be associated with an increased risk of renal cell carcinoma (Steenland and Woskie, 2012; Consonni et al., 2013). Although the evidence of the exposure response relationship is limited, it is still suggestive. In the study on the incidence rate in worker cohorts, the exact association between renal cell carcinoma and PFOA could not be shown (Steenland et al., 2015). The possible reason is that there were too few cases of renal cell carcinoma among workers, but there were positive findings in the community and worker joint cohorts.

The C8 scientific team also found suggestive evidence in its community and worker cohort study (Steenland and Woskie, 2012; Barry et al., 2013; Vieira et al., 2013). In three studies on drinking water polluted by PFOA in the United States, Sweden and Italy, the results showed that higher serum levels of PFOA may be related to renal cancer (Vieira et al., 2013; Mastrantonio et al., 2018; Li et al., 2022c). The Italian Ecological Mortality Study found that the death rate of renal cancer in PFAS-exposed areas was higher, and the RR of female renal cancer was significantly higher than 1.0. Although most studies showed that the risk of male renal cancer was high, Mastrantonio et al. reported that the risk of female renal cancer was higher (Mastrantonio et al., 2018). Researchers from the National Cancer Institute conducted a nested case-control study on 324 cases of renal cell carcinoma (RCC). After adjusting for a number of chemicals, they found that only PFOA was significantly associated with renal cell carcinoma (Shearer et al., 2021). The researchers reported that such results were probably attributable to reverse causality caused by the decline of renal function in RCC cases, but further adjustment for potential confounding factors such as obesity and hypertension, greatly increased the weight of the evidence that PFOA was a renal carcinogen. Because this is the largest investigation on PFOA exposure and RCC risk so far, and it is also the first study to use prospective examination of serum PFAS concentration before diagnosis for correlation with RCC. It is worth noting that the exposure of the general population in this study was much lower than that of other study populations. Another meta-analysis on renal cancer and testicular cancer showed that every 10 ng/mL increase in serum PFOA of patients with renal cancer significantly increased cancer risk, and these associations were most likely causal relationships (Bartell and Vieira, 2021).

**FIGURE 3:**
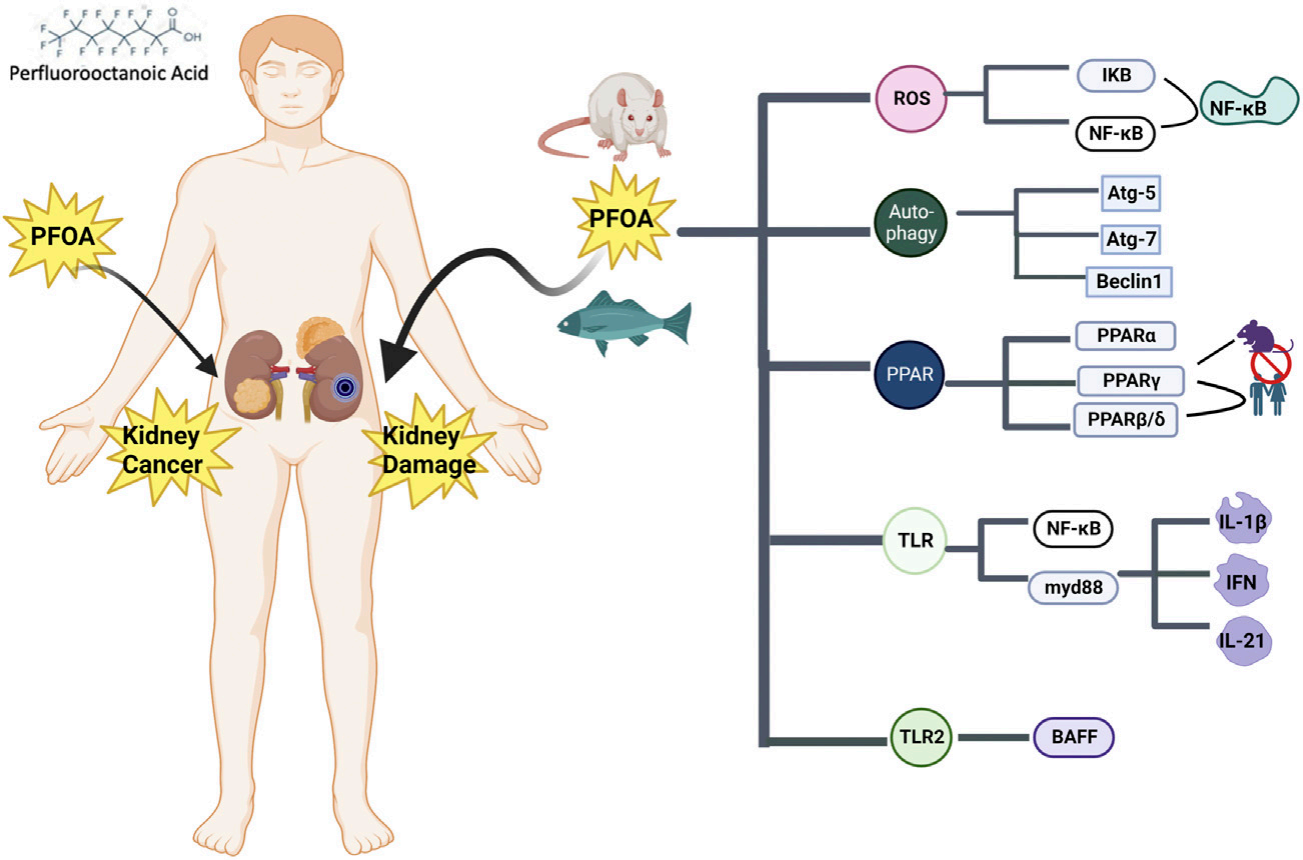
Effects of perfluorooctanoic acid on the kidneys and mediated mechanisms.

The International Agency for Research on Cancer (IARC) has classified perfluorooctanoic acid (PFOA) as a possible human carcinogen (Group 2B), partly because of limited epidemiological evidence related to renal cancer (IARC, 2017). Thus far, the evidence base of PFOA and renal cell carcinoma is still sparse. The relationship between PFOA and renal cell carcinoma is possible, the evidence is sufficient to conclude that PFOA is a possible cause of renal cancer (Steenland et al., 2022) (Figure 3).

### 4.1 Perfluorooctanoic acid (PFOA) and signaling pathways associated with kidney injury

In some studies, PFOA exposure altered several pathways related to kidney disease, including oxidative stress pathway, peroxisome proliferator activated receptor pathway, inflammatory pathway, autophagy pathway and DNA methylation. It is reported that PFOA causes oxidative stress in the kidney and liver of experimental animals, and is a reasonable mechanism of PFOA toxicity (Cui et al., 2009). When the oxidation of ROS is stronger than the antioxidant reaction of the cell defense system, oxidative stress will occur. It usually determines the peroxidation of membrane lipids, DNA damage and mutation, the oxidation and inactivation of proteins and enzymes, and the activation of apoptosis or necrosis processes (Bonato et al., 2020). In order to study the effect of PFOA on liver and kidney function of rats, the authors used N-acetylcysteine (NAC is a typical antioxidant). The results showed that NAC significantly reduced the increase of biomarkers of liver and kidney function toxicity induced by PFOA. The increase of oxidative stress and lipid peroxidation in rats treated with PFOA was restored to normal by NAC, and the proinflammatory mediator was reduced (Owumi et al., 2021). Another study explored the cytotoxic mechanism of lymphocytes isolated from the head and kidney of crucian carp after exposure to PFOA for 12 h. The results showed that PFOA caused oxidative damage to the lymphocytes of crucian carp, and led to the imbalance of various autophagy signal pathway related genes in the lymphocytes (Tang et al., 2018).

Environmental pollutants such as perfluorooctanoic acid (PFOA) can activate PPAR and by activating PPARα (Nuclear receptor peroxisome proliferator α) to change kidney function, but the correlation between human exposure and this mode of action is questioned. PPARα is expressed in liver and kidney (Klaunig et al., 2003; Andersen et al., 2008). PPAR receptor family includes three main homotypes, PPARα, PPARβ and PPAR γ. Research shows that PPARα mRNA is highest in adrenal gland and kidney (Abbott et al., 2012). Salmon continuously exposed to dietary PFOS and PFOA showed changes in peroxisome reaction and oxidative stress reaction, and the degree of kidney damage was higher than that of liver. The data showed that ROS mediated oxidative damage may be the major toxic mechanism of PFOA on fish, and after exposure to PFOA, PPARα and PPARγ mRNA showed organ-specific expression differences in liver and kidney, both of which were related to ACOX1 expression (Arukwe and Mortensen, 2011). Research in which activation of mouse and human peroxisome proliferator activated receptors by perfluorooctanoic acid and perfluorooctane sulfonic acid (α/β/δ/γ) showed that PFOA could activate mouse and human PPAR α and mouse PPAR β/δ. However, PFOA was not observed to significantly activate PPAR γ in mice or humans (Takacs and Abbott, 2007).

Another research study in which immunotoxicity induced by perfluorooctanoic acid in zebrafish kidney showed that PFOA can regulate the transcription of cytokines by affecting the transcription factors of the NF-κB family members. PFOA may initially attack toll-like receptor (TLR) and affect MyD88 and NF- κ B pathways to regulate cytokine transcription and induce zebrafish immune system imbalance (Zhang et al., 2021). After acute oral exposure to PFOA, epigenetic changes (including DNA methylation and HDAC expression) in mouse kidney tissue and kidney promote the expression of early markers of fibroblast activation (Rashid et al., 2020).

## 5 Discussion

Complex environmental chemicals such as PFAS are usually divided into compounds with different chain lengths for research. The damage to the human body and the main accumulation sites of each individual chemical are also different (Pelch et al., 2019). As a prominent environmental persistent organic pollutant, the harm of PFOA to kidney health has been reflected in this paper. The results of PFOA exposure to humans reported in the published literature are varied. Since the kidney is the target organ of PFOA, and the elimination from kidney is the main elimination pathway of PFOA, the harm is more prominent (Han et al., 2012). In this article, we summarized the literature on the relationship between PFOA exposure and pathological changes of renal function. Although we have described many possible mechanisms of PFOA underlying renal tissue or cytotoxicity, the mechanism of PFOA on renal damage has not been fully understood, and further research is needed. For example, PPARα is a suspected nuclear receptor target of PFAS, which is expressed in the kidney. However, the degree of PPARα activation by PFAS is considered to vary depending on the carbon chain length and functional groups. Some PFAS showed a high level of PPARα activation (e.g. PFOA), while others did not (e.g. PFDeA, perfluorodecanoate) (Wolf et al., 2008). The signaling pathway is mostly tested on fish and mice, and conclusions from human cell culture is lacking, which needs further verification. ROS homeostasis, NF-κB activation and PPARα are the key nodes and needs for further verification in the future.

Due to the reabsorption of the kidney and the continuous emission of PFOA pollutants in the living environment, PFOA has a long half-life in the human body. Therefore, perfluorooctanoic acid will continue to cause damage to human health (Post et al., 2012). The exposure of perfluorooctanoic acid is ubiquitous for human beings, so this exposure is added to all other exposures that human beings may experience, including the co-exposure with other environmental pollutants, such as heavy metals, other PFAS, etc., which aggravates the problem of possible mixture effects, and even produces synergistic and enhancement effects (Jensen and Leffers, 2008). In this review, we present some new findings. The NHANES data from 2003 to 2014 were used to evaluate the impact of the joint exposure of toxic metal cadmium, lead, mercury and perfluoroalkyl substances on healthy renal function. It was found that the adjusted odds ratio of healthy renal function were 0.657 and 0.671 respectively when exposed to high levels of lead, high levels of PFOA and PFNA at the same time (odds ratio less than one indicates unhealthy renal function) (Jain, 2019). Not only environmental pollutants, but also the joint exposure of PFOA and disease will aggravate renal injury. *In vitro* experiments show that under the condition of diabetes, after treatment of rat mesangial cells with PFOA, the expression levels of oxidative stress, fibrosis and proinflammatory markers in cells further increase, indicating that perfluorooctanoic acid may aggravate renal injury in animals or humans (Gong et al., 2019). These findings provide a new direction for future research on the adverse effects of PFOA and other exposures on kidneys.

In addition, PFAS also has the potential of non-monotonic dose response curves. Low concentration exposure may have more destructive effects than high concentration exposure, especially related to the endocrine system, such as thyroid function or puberty development (Rappazzo et al., 2017). It is worth noting that contaminated food, drinking water, air and dust are the main sources of PFOA exposure for non-occupationally exposed people. Not all people are equally affected by PFOA, and exposure may depend on work, age, sex, exposure time and exposure dose (Holzer et al., 2008; Toms et al., 2009). These differences bring many difficulties to the study of PFOA on renal injury. Therefore, focusing on the effects of PFOA on renal function of different species, different age groups and different exposed people will help to explore the mechanism of PFOA in the future and provide more theoretical reference for the related research on PFOA-induced renal injury.

Thus far, the Environmental Protection Agency (EPA) estimates that there are more than 9000 different PFAS compounds, including parent PFAS and its metabolites and degradation products (Baker and Knappe, 2022). Although some countries such as the United States have banned the production of PFOA and PFOS (USEPA, 2006), developing countries such as China are still producing them in large quantities. It is logical to expect continued exposure to perfluorinated compounds in the future (Huang et al., 2022; Jiao et al., 2022). Therefore, it is necessary to properly regulate PFAS such as perfluorooctanoic acid (De Silva et al., 2021). At the same time, it is necessary to fill a large number of gaps that still exist, including the toxicological information and toxicity mechanism of PFAS such as perfluorooctanoic acid or its emerging substitutes (Jane et al., 2022). There may be thousands of PFAS that have not been tested, and some of them lack toxicity, which does not mean that other PFAS are safe. Therefore, it is more important to determine whether emerging PFAS or PFAS substitutes may have more far-reaching renal effects than other PFAS, and link them with the understanding of renal function and toxicity mechanism. Finally, in order to better understand the impact of PFAS on the kidney, it is expected that future research can better link the accumulation of PFAS with the kidney impact of mothers and offspring, so as to fill a large number of gaps currently existing.

Our research has some advantages: First of all, perfluorooctanoic acid, as a new environmental pollutant, has been rarely studied, especially the review on PFOA and kidney. Our research provides relevant experience for reference. Secondly, we comprehensively reviewed the relationship between perfluorooctanoic acid and kidney, including animal experiments, cell experiments and epidemiological investigations, summarized the possible effects of PFOA on renal function (uric acid, glomerular filtration rate) and renal diseases (chronic kidney disease, renal cancer), and discussed the toxicological mechanism related to renal injury, so as to better understand the relationship between the two.

Of course, our research also has some limitations: First, most studies on the relationship between PFOA and kidney are cross-sectional studies, and the evidence is limited. More epidemiological studies are needed to verify the results. Second, our research is limited to discussing the relationship between PFOA and kidney, and it is expected that future research will better explore the relationship between other PFAS and human organs in the future.
